# Vitamin K as a Diet Supplement with Impact in Human Health: Current Evidence in Age-Related Diseases

**DOI:** 10.3390/nu12010138

**Published:** 2020-01-03

**Authors:** Dina C. Simes, Carla S. B. Viegas, Nuna Araújo, Catarina Marreiros

**Affiliations:** 1 Centre of Marine Sciences (CCMAR), University of Algarve, Campus de Gambelas, 8005-139 Faro, Portugal; caviegas@ualg.pt (C.S.B.V.); naraujo@ualg.pt (N.A.); cimarreiros@ualg.pt (C.M.); 2 GenoGla Diagnostics, Centre of Marine Sciences (CCMAR), University of Algarve, Campus de Gambelas, 8005-139 Faro, Portugal

**Keywords:** vitamin K, diet supplement, age-related diseases, vitamin K-dependent proteins, pathological calcification, inflammation

## Abstract

Vitamin K health benefits have been recently widely shown to extend beyond blood homeostasis and implicated in chronic low-grade inflammatory diseases such as cardiovascular disease, osteoarthritis, dementia, cognitive impairment, mobility disability, and frailty. Novel and more efficient nutritional and therapeutic options are urgently needed to lower the burden and the associated health care costs of these age-related diseases. Naturally occurring vitamin K comprise the phylloquinone (vitamin K1), and a series of menaquinones broadly designated as vitamin K2 that differ in source, absorption rates, tissue distribution, bioavailability, and target activity. Although vitamin K1 and K2 sources are mainly dietary, consumer preference for diet supplements is growing, especially when derived from marine resources. The aim of this review is to update the reader regarding the specific contribution and effect of each K1 and K2 vitamers in human health, identify potential methods for its sustainable and cost-efficient production, and novel natural sources of vitamin K and formulations to improve absorption and bioavailability. This new information will contribute to foster the use of vitamin K as a health-promoting supplement, which meets the increasing consumer demand. Simultaneously, relevant information on the clinical context and direct health consequences of vitamin K deficiency focusing in aging and age-related diseases will be discussed.

## 1. Introduction

Historically recognized as a key factor for the synthesis of blood clotting factors in the liver, vitamin K is currently known to be involved in a wide range of biological processes and is associated with many pathological conditions. Since its discovery in 1936 [1], the most well-known function of vitamin K is as a cofactor for the γ-glutamyl carboxylase (GGCX) enzyme responsible for the post-translational modification of vitamin K-dependent proteins (VKDPs) through the conversion of specific glutamic acid (Glu) into calcium binding γ-carboxyglutamic acid (Gla) residues [2,3]. In humans, at least 17 different VKDPs, which are also known as Gla proteins, have been identified to date, and are generally referred to as hepatic and extra-hepatic VKDPs, according to the synthesis location (Table 1). The hepatic group of VKDPs synthetized in the liver are essential for regulating blood coagulation and comprise the coagulation factors II, VII, IX, and X, and the anti-coagulation proteins C, S, and Z. Extra-hepatic VKDPs include matrix Gla protein (MGP), osteocalcin (OC), Gla-rich protein (GRP), growth arrest-specific protein 6 (Gas6), proline-rich Gla proteins (PRGP1 and 2), transmembrane Gla proteins (TMG3 and 4), periostin, and the GGCX enzyme. These extra-hepatic VKDPs, which are mostly known for their protective role in the bone and cardiovascular system, exhibit a broad tissue distribution and are involved in a wide range of biological functions such as bone homeostasis, ectopic calcification, cell differentiation and proliferation, inflammation, and signal transduction. γ-carboxylation has been shown essential for the correct function of VKDPs. In addition, vitamin K deficiency has been linked to several pathological conditions such as cardiovascular diseases (CVD), chronic kidney disease (CKD) [4], osteoarthritis (OA) [5], rheumatoid arthritis (RA), osteoporosis, cancer, dementia, certain skin pathologies, functional decline, and disability [6]. Most of these chronic health conditions are associated with pathological calcification and inflammation, where the role of VKDPs and vitamin K is being highlighted. Since both inflammation and pathological mineralization are associated with the aging process and these diseases are highly prevalent in the elderly, a new concept on the involvement of vitamin K in *inflammation* is growing. In addition, novel roles have been disclosed for vitamin K independent of its activity as a cofactor for GGCX, such as an antioxidant, anti-inflammatory, promoter of cognition, inhibition of tumor progression, and transcriptional regulator of osteoblastic genes. However, in clinical practice, vitamin K is mainly used in blood clotting-associated prophylaxis. The number of in vitro, in vivo, and clinical data showing the beneficial effects of vitamin K without adverse effects or documented toxicity raised increasing interest on the use of vitamin K as a health promoting supplement. In fact, aging societies represent a major economic challenge for health care systems, and diet supplements promoting healthy aging and improving the prognosis of age-related diseases, are required to be implemented in clinical practice.

In this context, it is crucial to highlight that naturally occurring vitamin K comprise the vitamin K1 (also known as phylloquinone or phytonadione), and a series of menaquinones (MKs), designated as vitamin K2 [3,6]. A growing amount of scientific evidence has demonstrated differences between vitamin K1 and K2 in terms of source, function, and target activity. While vitamin K1 and K2 from different sources are currently commercially available and are becoming popular as health supplements, novel tactics for more efficient and affordable attainment of both vitamers are currently being explored. However, it is important to clearly establish a specific cause-effect for each of the vitamin K vitamers to increase efficacy and disease-target specificity. 

This work thoroughly reviews available data regarding differences between vitamin K1 and K2, contextualized with clinical aspects of vitamin K deficiency, including their sources, functions, target activity, and involvement in age-related diseases. Processes for the chemical and biological production of vitamin K1 and K2 will be briefly addressed. Additionally, novel sources with potential biotechnological application, and new formulations to improve vitamin K absorption and bioavailability are presented.

## 2. Clinical Context of Vitamin K 

### 2.1. Vitamin K Deficiency

Vitamin K deficiency is clinically characterised by a bleeding tendency due to the loss of function of vitamin K-dependent hepatic clotting factors. Vitamin K deficiency is not very common in adults and is usually associated with specific conditions, such as malabsorption disorders, antibiotics, and drug interactions, especially with coumarin-based anticoagulants, or an extremely poor vitamin K-content diet. Anticoagulant treatment with coumarin derivatives is widely prescribed to prevent thromboembolic events or stroke in patients with atrial fibrillation or cardiac disease. Clinical evaluation of the vitamin K status is assessed only in specific conditions, such as individuals with bleeding disorders or under anticoagulants. 4-hydroxycoumarin anticoagulant drugs such as warfarin, acenocoumarol, and phenprocoumon are widely used oral anticoagulants acting as vitamin K antagonists (VKAs). In these cases, prothrombin time, which is indicative of the time necessary for blood clotting and known as the International Normalized Ratio (INR), is usually the parameter used for a drug dosage. Treatment with VKAs inhibits the recycling of vitamin K and the synthesis of the vitamin K-dependent biologically active clotting factors II, VII, IX, and X. Patients’ warfarin dose is adapted based on INR scores so that it remains in the therapeutic range to prevent thrombosis or haemorrhagic complications [34]. 

Gastrointestinal disorders can compromise the pancreatic/biliary functions and trigger fat absorptive mechanisms, such as in the case of celiac disease, cystic fibrosis, ulcerative colitis, cholestasis, short bowel syndrome, or in a situation of bariatric surgical intervention. These conditions might lead to a situation of inadequate absorption of vitamin K and, ultimately, to a status of vitamin K deficiency. 

Antibiotics also interfere with vitamin K levels since they generally contribute to a decrease of vitamin K-producing bacteria in the gut [35]. Vitamin K deficiency is currently observed in patients with prolonged oral broad-spectrum antibiotic therapy. Antibiotics such as cephalosporins, which include the N-methylthiotetrazole side chain, are suggested as inhibitors of hepatic vitamin K epoxide reductase [36,37,38]. A nested case-control study performed in a cohort of 6191 patients concluded that patients receiving cephalosporins and other antibiotics for more than 48 h had an increased risk of haemorrhagic events [39]. 

Some medications are also reported to interfere with vitamin K absorption. These are drugs prescribed to reduce cholesterol in a dyslipidemia scenario, or drugs that interfere with lipases activity used for obesity treatment such as orlistat, or bile acid sequestrants, such as colesevelam and cholestyramine. Overall, these medications can affect the absorption of fat-soluble vitamins and lead to a decrease in the vitamin K status [40,41]. In these situations, monitoring and supplementation might be recommended.

Newborns might experiment with vitamin K deficiency during the first few weeks of life as a consequence of a combination of factors that contribute to low levels of vitamin K [42]. Poor vitamin K carriage through placenta, low content of vitamin K1 in breast milk, and liver immaturity that leads to an inefficient use of vitamin K and recycling represent part of the conditions that could lead to a situation known as vitamin K deficiency bleeding (VKDB) [43,44]. To reduce the incidence of VKDB, oral vitamin K prophylaxis with phytomenadione, or administration of a single intramuscular (IM) dose of 0.5–1 mg at birth is recommended by the World Health Organization (WHO) [45]. 

In the last decade, the bulk of research on vitamin K has shifted beyond coagulation, which further explores its physiological role in skeletal, CVD, and brain health [46,47]. Vitamin K deficiency has been associated with a higher risk of age-related chronic diseases such as osteoporosis, CVD, RA, and OA, which contributes to its onset and progression [5,48,49,50]. Several extra-hepatic VKDPs such as MGP, GRP, and OC are well described to be of vital importance in the pathophysiology of these age-related diseases. More recently, several in vitro and in vivo studies, as well as clinical data, have highlighted the role of vitamin K in cognitive performance, particularly associated with Alzheimer’s disease. The action of vitamin K in brain cells’ development and survival has been linked with its role in the synthesis of sphingolipids, and through the function of the VKDPs, Gas6, and protein S [51]. Although the association between vitamin K deficiency and cognitive impairment is still not definitively established, several works have shown a direct correlation between low levels of vitamin K and deterioration of cognitive and behavioural performances (recently reviewed in Reference [47]). 

In fact, the current use of oral anticoagulants acting as VKAs, has also been associated with adverse clinical outcomes in extra-hepatic tissues such as bone, cartilage, the vascular tree, and brain [52,53,54,55].

### 2.2. Vitamin K Antagonists (VKAs)

The role of vitamin K in arterial calcification has been associated with its function as a co-factor for carboxylation of MGP and GRP. The role of MGP as an inhibitor of vascular calcification (VC) is strictly dependent on its γ-carboxylation status [56]. In addition, only the carboxylated form of GRP was shown to have calcification inhibitory properties [26]. Preclinical studies clearly demonstrate that the use of VKAs induces a vitamin K deficiency status, which enhances medial and intimal calcification in the vascular tree. Vitamin K treatment was shown not only to be able to inhibit mineralization in warfarin-treated rats, but even to promote the regression of the pre-formed medial elastocalcinosis [57]. This is in line with several studies demonstrating negative effects of warfarin treatment in cardiovascular health [58]. In humans, the detrimental effect of VKAs on extra-coronary calcification has been shown on a small cross-sectional study in patients on long-term oral coumarin treatment [59]. In another cohort study assessing 430 patients, the presence of calcification in peripheral arteries was compared between warfarin patient users and non-users. In this study, the prevalence of arterial calcification was 44% greater in patients on warfarin therapy versus without warfarin use [60]. 

Several [61,62,63] clinical studies, but not all [64] clinical studies, give indications that warfarin promotes atherosclerotic calcification, since an increase in coronary calcification, which is predominantly atherosclerotic, was observed in patients using VKAs. An observational study reported that the use of VKAs is related with increased aortic stiffness in end-stage CKD patients undergoing haemodialysis (HD) [65]. In a retrospective clinical study including patients on haemodialysis who developed calciphylaxis, 6/8 patients were on VKAs therapy. This suggests VKAs therapy as one of the factors involved in the development of calciphylaxis [66], which is a rare but fatal complication in CKD patients, characterized by ischemic skin ulceration due to mineralization affecting subcutaneous small arterioles. 

Recently, a large population-based cohort study including individuals from the Gutenberg health study comprising 287 VKAs users and 14,564 VKAs non-users demonstrated that patients on VKAs therapy had a higher cardiovascular burden. In this cross-sectional study, although no cause-effect interpretation could be made, the authors suggest a relation between VKAs use and several parameters of clinical and subclinical CVD, such as with increased arterial stiffness, decreased cardiac systolic function, and higher left ventricular mass. This study also shows an association and dose-response effect of VKAs intake with low grade systemic inflammation. This was shown by the high levels of high-sensitivity C-reactive protein (hsCRP) found in long-term VKAs users when compared with short-term VKAs intake users [67]. A post hoc analysis used serial coronary intravascular ultrasound examinations and involved eight prospective randomized trials. Changes in coronary atheroma burden were compared between patients with coronary artery disease patients treated with (n = 171) and without (n = 4129) warfarin for 18 to 24 months. In this study, the authors concluded that warfarin use was independently associated with serial coronary calcification with no association with renal function, statin therapy, or changes in atheroma volume [68].

Overall, the available studies and the information on the detrimental side effects of VKAs reinforces the notion that special care should be given on their clinical use. This is even more relevant for patients requiring long-time anticoagulant therapy and for those considered to be at higher atherosclerotic risk. Furthermore, new direct oral anticoagulants (DOACs) drugs that do not inhibit vitamin K recycling and target different factors in the coagulation cascade, specifically factor Xa and thrombin, such as dabigatran, rivaroxaban, apixaban, and edoxaban, are now available as medications. More recently, a cross-sectional observational study including 236 atrial fibrillation patients were divided in three groups, according to the type of anticoagulation therapy (no oral anticoagulation, VKAs or DOACs). The main findings of this study pointed toward an increased prevalence of calcification of the thoracic aorta in patients treated with VKAs when compared to patients with DOACs treatment, with no effect on calcification observed in DOACs-treated patients compared with no oral anticoagulation group [69]. 

DOACs are recognized as an attractive alternative option to VKAs for short-term and long-term coagulation due to their single-dose oral administration without the need for repeated blood monitoring, their proven safety, and short half-life [70]. This will allow the safe use of vitamin K as a supplement to prevent soft tissue pathological mineralization in aging diseases such as osteoporosis, OA, CKD, and CVD. Nevertheless, randomized controlled trials addressing causality of VKAs and DOACs on VC are still warranted. 

## 3. Vitamin K1 and K2: Similar Function but Different Absorption, Storage, Bioavailability, and Targets

### 3.1. Vitamin K Chemical Structure

**Figure 1:**
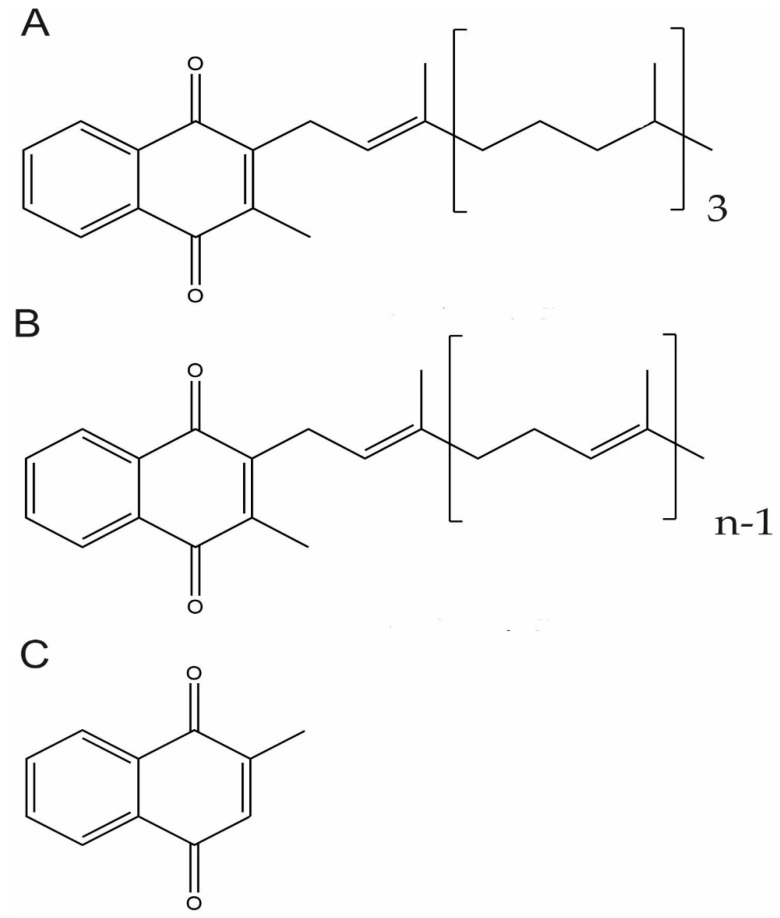
Chemical Structure of Vitamin K vitamers. Phylloquinone or vitamin K1 (**A**), menaquinone-n (MKn), or vitamin K2 (**B**) and menadione or vitamin K3 (**C**).

Vitamin K are fat-soluble vitamins that occur and function in the membranes of living organisms and comprise vitamin K1 and vitamin K2. Both forms share a 2-methyl-1,4-naphtoquinone double ring structure in their chemical backbone (menadione, vitamin K3) but differ in their lipophilic side chain (Figure 1). While vitamin K1 has a phytyl substituted chain, vitamin K2 contains unsaturated isoprenyl side chains, designated as MK-4 through to MK-13, depending on its length [71,72]. Vitamin K1 is present in vegetables, mainly in green leafy vegetables, vegetable oils, and some fruits and it is the main source of vitamin K in diet. Vitamin K2 is found in animal-based and fermented foods or produced by bacteria in the human gut. MK-4 is an exception since is not a common product of bacterial synthesis but considered to be of animal origin based on its tissue-specific conversion from vitamin K1 [73,74]. On the other hand, although often referred to as vitamin K3, menadione is not a natural component of foods but is considered a product of catabolism of vitamin K1 and a circulating precursor of tissue MK-4 [72,74]. For this reason, it should, more adequately, be known as a pro-vitamin.

### 3.2. Vitamin K Functions

Both vitamin K1 and K2 can act as cofactors in the carboxylation process of VKDPs. The reduced form of vitamin K (vitamin K hydroquinone, KH_2_) is the active cofactor for the γ-glutamyl carboxylase (GGCX) enzyme, which modifies Glu residues to Gla residues in VKDPs. The reaction also requires carbon dioxide and oxygen. During the carboxylation reaction, vitamin K 2,3-epoxide (KO) is continuously recycled by vitamin K epoxide reductase (VKOR) and vitamin K reductase (VKR), to its quinone (K) and KH_2_ forms in a process known as the vitamin K cycle [75,76,77]. Due to this efficient cell recycling process, the organism can preserve limited nutritional stores of vitamin K depending on minimal vitamin K amounts to cover its daily diet requirements [78]. Of note, a higher bioactivity of MK-7 relative to K1 as a cofactor of GGCX-mediated protein carboxylation in both hepatic and extra-hepatic tissues is supported by several in vitro and in vivo studies. An in vitro study demonstrated that cofactor activity of vitamin K increased with the length of the aliphatic side chain [79]. 

In addition to the proposed involvement of some VKDPs in inflammation processes, vitamin K has been proposed to act as an anti-inflammatory and antioxidant agent independent of its GGCX cofactor activity. Several in vitro and animal studies have shown that vitamin K reduced the activation of nuclear factor kappa B (NFκB) and inhibited IkappaB kinase (IKB) α/β phosphorylation, with a consequent decrease in the production of pro-inflammatory cytokines [80,81,82]. This action was proposed to be mediated through the naphthoquinone ring of vitamin K, and it is not surprising that both vitamin K1 and K2 (MK-3, MK-4, and MK-7) were found to suppress a liposaccharide (LPS)-induced inflammatory state in vitro and in vivo in the mouse model. In addition, a role for vitamin K as an antioxidant agent has been proposed. The reduced form of vitamin K (KH_2_) was shown to protect phospholipid membranes from peroxidation by direct reactive oxygen species (ROS) uptake [83,84]. An important player in this antioxidant activity is the paralogous enzyme of the vitamin K epoxide reductase complex subunit 1 (VKORC1), which is the vitamin K epoxide reductase complex subunit 1 (VKORC1)-like 1 (VKORC1L1), responsible for increasing KH_2_ intracellularly and limiting the amount of intracellular ROS [85]. In cultured neurons and oligodendrocytes, vitamin K was shown to prevent cell death caused by oxidative stress by inhibiting the activation of 12-lipoxygenase (12-LOX). Both vitamin K1 and K2 have been shown to have antioxidant properties [86,87]. Recently, a study aiming to evaluate the effect of vitamin K on the redox metabolism of human osteoblasts cultured in the presence of hydroxyapatite-based biomaterials showed that vitamin K prevented a redox imbalance by decreasing ROS levels. The highest effect was obtained with MK-7 [88]. 

The involvement of vitamin K with sphingolipids metabolism, although known for some decades, has recently gained renewed attention due to suggested implications of alterations in sphingolipid metabolism with the aging process [89] and neurodegenerative disorders such as Alzheimer’s and Parkinson’s diseases [90,91]. It has been shown that vitamin K activates 3-ketodihydrosphingosine (3-KDS) synthase (also known as serine palmitoyltransferase), which is the enzyme involved in the initial step of sphingolipid biosynthesis [92], and the sulfotransferase responsible for sulfatide synthesis [93]. In rats, warfarin treatments were associated with decreased activity of 3-KDS synthase and sulfotransferase, and significant reductions in brain sulfatides, sphingomyelin, and cerebrosides [93,94]. Data on the association of specific vitamin K vitamers with sphingolipid metabolism in humans is still scarce and warrants further investigation. The predominant form of vitamin K in the brain of rats and humans is MK-4 [95,96]. In rats, the stimulatory effect of vitamin K on the activity of sphingolipids metabolism enzymes was observed with either K1 or MK-4 as a source of vitamin K [97]. In addition, the concentration of MK-4 in the rat brain was shown to positively correlate with the concentration of sphingolipids, particularly with sulfatides and sphingomyelin, and both K1 and MK-4 increased with K1 intake [96]. This is in line with our knowledge that MK-4 is a result of K1 conversion, and that cerebral MK-4 originates from K1 intake [98]. In humans, a few studies have shown a relationship between low levels of K1 and Alzheimer’s disease and impaired memory performance in older adults [99,100]. However, specific relationships between higher K1 or MK-4 levels and sphingolipid synthesis requires further elucidation. A currently active field of research around vitamin K relates to its potential anti-cancer effect. Although this topic will not be explored in this review, vitamin K has been implicated with the inhibition of several neoplastic cell lines mainly by inducing apoptosis and cell cycle arrest of cancer cells through various mechanisms [101]. Among the different forms of vitamin K tested, vitamin K2 was shown to inhibit several cancer cell lines without side effects and has been selected as a promising agent for cancer prevention and clinical therapy. Clinical trials have demonstrated the potential of vitamin K2 to improve the prognosis of cancer patients [102,103]. 

### 3.3. Vitamin K1 and K2 Absorption, Storage, and Bioavailability

Although both vitamin K1 and K2 are involved in γ-carboxylation of VKDPs, these molecular forms act differently in processes such as absorption, transport, cellular uptake, tissue distribution, and turnover [78]. Despite sharing a similar structure (Figure 1) and physicochemical characteristics, natural vitamin K forms have different lipophilicity. The longer-chain menaquinones, including MK7, are much more hydrophobic and have longer half-times. Although there are many studies reporting different results, it seems clear that the length and degree of saturation of the isoprene side chain influences their clearance from circulation and bioavailability [72]. In healthy adults, absorption of MKs (MK-4, MK-7, and MK-9) has been compared with K1. The results indicate that MK-7 is the most efficiently absorbed form of vitamin K [104,105,106]. Although K1 is the major type (>90%) of dietary vitamin K, it is poorly retained in the organism. Its concentrations in animal tissues are remarkably low when compared with those of MKs, especially MK-4, which is the major form (>90%) of vitamin K found in animal tissues [74,107]. 

Both vitamin K1 and K2 forms follow a similar and well-established intestinal absorption pathway. Following their packing into chylomicrons, they are further transported in circulation to their target tissues by lipoproteins [78,106]. While vitamin K1 in circulation is mostly associated with triacylglycerol-rich lipoproteins (TLR), vitamin K2 is mainly transported by low-density lipoproteins (LDL). This difference could also justify the higher half-life time, bioavailability, and higher bioactivity of MK-7 when compared with vitamin K1. A study comparing vitamin K1 and MK-7 shows that MK-7 had a half-life time of 68 h compared with only 1–2 h for K1 [105]. This results in more stable blood levels and a higher bioavailability of MK-7, while vitamin K1 is rapidly removed from circulation, accumulated in the liver, and excreted in urine and bile. In the Japanese population, known for its higher K2 diet intake, mainly due to natto consumption, MK-7 was found to be the predominant circulation form of vitamin K [108]. The concept that long-chain MKs are available longer in circulation than K1 for cell uptake supports the suggestion that vitamin K2 represent a more adequate form of vitamin K delivery to extra-hepatic tissues such as bone and the vasculature. In fact, using equimolar amounts of both vitamin K forms as supplements, a cross-over study showed that circulating levels of carboxylated OC were higher in subjects taking MK-7 when compared to the vitamin K1 supplemented group [105]. In the same study population, another cross-over study shows that MK-7 was almost three times more potent than K1 in counteracting the effect of coumarin anticoagulants [105]. This rationale might explain the reported prevalent association of vitamin K2 and not K1 intake, with a reduced risk of CVD [109]. In fact, although several studies have demonstrated a relation between vitamin K1 and cardiovascular health, studies aiming to compare the effects of K1 and K2 clearly highlight the prevalence of K2 as a cardiovascular protective agent. In the prospective, population-based Rotterdam Study, comprising 4807 subjects free from myocardial infarction at baseline, followed up for 7 years, low levels of vitamin K2 but not K1 were associated with a significant risk in coronary heart disease (CHD), all-cause mortality, and severe aortic calcification [109]. In the Prospect-EPIC cohort study, enrolling 16,057 women free from CVD at baseline, with a mean follow-up of 8.1 years, an inverse association between vitamin K2 (particularly MK-7, MK-8, and MK-9) and risk of CHD was found with an 85%–100% reduction in coronary events for every 10 µg increase in vitamin K2 intake [110]. Again, vitamin K1 intake was not significantly associated with cardiovascular outcomes [110,111].

In relation to bone health, although vitamin K2 has been suggested as the vitamer with the highest bone-protecting effects, available clinical data is still conflicting in this subject. In fact, the effect of vitamin K supplementation has been evaluated in several clinical trials using either K1 or K2, with results pointing for a protective effect of both vitamers through the improvement of bone quality with increased strength and reduced turnover, and a reduction in fractures (reviewed in Reference [6]). Several inconsistent results are found in the literature concerning the specific effects of each vitamin K vitamer. This might be explained by the small sample number in interventional studies and the heterogeneity associated with these studies specifically related to different evaluation methods for vitamin K status, supplementation doses, and specific types of vitamin K. Importantly, simultaneous comparisons between the effects of K1 and different MKs such as MK-4 and MK-7 on bone outcomes should help the clarification of the most suitable vitamer for improving bone health. 

## 4. Dietary Sources of Vitamin K1 and K2

Vertebrates, including humans, do not synthesise vitamin K and depend on dietary sources to obtain the required daily allowance. Moreover, vitamin K body storage is rapidly depleted in the absence of a regular dietary intake [112]. Comprehensive reviews addressing both vitamers content in a variety of foods have recently become available [104,113,114,115]. However, only a few national food composition databases including vitamin K content are available, and most of them do not include specific information on K1 and K2 content in each food item.

Vitamin K1 is a final product of the shikimate pathway in the photosynthesis process, and, therefore, can be found in all photosynthetic organisms, including plants, algae, and cyanobacteria [116,117]. The main sources of dietary vitamin K are green leafy vegetables such as kale, romaine lettuce, broccoli, cabbage, and spinach [115]. Vegetable oils such as soybean, sunflower, olive, and canola are the next best dietary source of K1 [118,119]. Lower amounts of K1 can also be found in fruits, cereals, meat, and dairy products [120]. High levels of vitamin K1 can be found in common Japanese food items such as in vegetables, with the highest value found in perilla (raw, 1007 μg/100 g), in edible seaweed such as hijiki (*Sargassum fusiform*, dried, 175 μg/100 g) and wakame (*Undaria pinnatifida* dried, 1293 μg/100 g) [121]. Different vitamin K contents have been reported for the edible red algae *Porphyra* sp., commonly known as laver or nori, describing levels of around 2600 μg/100 g on a dry basis in the dried nori, with a significant reduction found in toasted dry nori (approximately 390 μg/100 g on a dry basis) [122] and in roasted and seasoned laver (dried 413 μg/100 g) [121]. Additionally, different types of vegetable fats and oils such as soybean oil (234 μg/100 g) and green powdered tea (3049 μg/100 g), which are widely consumed in Japan, are reported to contain high amounts of K1 [108].

Vitamin K2 is mainly produced by bacteria, except for MK-4, which can be produced by tissue-specific conversion from vitamin K1 in animals. This reaction is catalysed by the UbiA prenyltransferase domain-containing 1 enzyme [74], which involves the menadione form as an intermediate. In fact, MK-4 formed from vitamin K1 can be found in higher amounts in animal organs not commonly consumed in the diet (liver, brain, pancreas, or kidney) [95]. Vitamin K2, such as MK-7, MK-8, and MK-9, which is the most recognized forms in terms of nutrition value [123], are biosynthesized by several obligate and facultative anaerobic bacteria [113,124]. In addition, the bacterial flora in the human gut is described to produce several long-chain MKs. In the human large intestine, the major forms of K2 found to be present, including MK-6, MK7, MK-8, MK-10, and MK11, are produced by several types of enterobacteria such as *Bacteroides, Enterobacteria*, *Eubacterium lentum*, and *Veillonella* [125,126]. Although intestinal bacteria synthesis is described to contribute to vitamin K requirements [127], it is not yet clear its true contribution to human vitamin K2 nutrition, and there is a need for further progress in this area [123].

The use of bacteria in food production processes has greatly increased in the last decade [128] along with the interest in the production of food products enriched with vitamin K2. Several lactic acid bacteria commonly used for making fermented food products, and generally recognized as safe (GRAS), have been used for the biosynthetic production of MKs for the last few decades, with significant production amounts of MKs (MK-7 to MK-10) [129]. Nevertheless, some genera of bacteria widely used in the food industry, including *Lactobacillus* and *Streptococcus*, have lost the functional ability to produce vitamin K2. Due to this, the K2 content of food products using these bacteria is almost undetectable [130]. A study examining the capacity of several bacterial strains to produce K compounds selected three strains of *Lactococcus lactis ssp. cremoris*, two strains of *Lactococcus lactis ssp. lactis*, and *Leuconostoc lactis* as high producers able to deliver more than 230 nmol/g dried cells of MK-7 to MK-10 [129]. In fact, several other bacterial species including *Brevibacterium linens, Brochontrix thermosphacta, Hafnia alvei, Staphylococcus xylosus, Staphylococcus equorum*, and *Arthrobacter nicotinae,* which are commonly used in industrial food fermentations, are well-known to produce several forms of K2, from MK-5 to MK-9, in different amounts [113].

Other major sources of vitamin K2 are meat, especially chicken, bacon, and ham [120]. In addition, egg yolks and high-fat dairy products, such as hard cheeses, provide appreciated amounts of this vitamer [73]. Of note, cheese was found to be the most important source of dietary long-chain MKs (MK-8 and MK-9) [131]. In particular, propionibacteria-fermented cheese, such as Norwegian Jarlsberg cheese and Swiss Emmental cheese, were shown to have the highest concentration of vitamin K2 in the form of tetrahydromenaquinone-9 [132]. Another important dietary source of vitamin K2, with interest for the industry, are fermented plant foods, such as natto. Natto is a traditional Japanese soybean food produced by fermenting cooked soybean with *Bacillus subtilis natto* and considered one of the most relevant dietary sources of MK-7 (around 1000 μg/100 g natto) [104,121,133].

## 5. Vitamin K1 and K2 Chemical and Biotechnological Production Methods

The health benefits of vitamin K [46,134], together with the growing trend for sustainable and natural health products, has led to a high interest on the search for sustainable and cost-effective processes to produce natural vitamin K. Both chemical and biochemical synthetic strategies for vitamin K are currently being explored. Chemical synthesis processes for both vitamin K forms, either vitamin K1 or vitamin K2, have been developed in the past few years [135,136,137], while biosynthetic production methods have been mostly explored for MKs (MK-4/MK-7). In addition, the continuous discovery of vitamin K vitamers in aquatic organisms, widely recognized as a valuable source of bioactive compounds and with biotechnological potentialities, might open new perspectives for novel vitamin K sources and production methods.

### 5.1. Vitamin K1

Currently, a chemical synthesis process for vitamin K1 is well established [138,139] and used in a wide range of commercial applications, from human nutrition to pharmaceutical products [140,141,142], but not in cosmetics where vitamin K1 formulations were banned from use in 2009 [143]. Improvements on the chemical synthesis methods of vitamin K1 have been mainly focused on the reduction of the inactive Z-isomer formation [141], and in decreasing the use of toxic chemicals that are hazardous to both the environment and humans [142,144]. One of the major chemical synthesis challenges resides in eliminating menadione traces [138,144]. In fact, menadione and its derivates such as menadione sodium bisulfite and menadione sodium diphosphate were banned from human products since 1963 due to evidence of toxicity, even though they are still used for animal feed as pro-vitamins [145]. Research on the biotechnological production of vitamin K1 is still quite incipient but constitute a promising route to increase the quality of the final product (active trans(E)-isomer), and to reduce the costs associated with the inclusion of this vitamin in diet supplements [146].

### 5.2. Vitamin K2

Chemical synthesis of vitamin K2, although described almost 40 years ago [147], remains challenging due to the need of stereoselective synthesis of the bioactive all-trans configuration. Despite traditional high cost and low yield, chemically synthetized vitamin K2 has gained renewed interest with new and optimized methods for an efficient and stereoselective production of high pure (99.9%) all-trans vitamin MK-7 with a moderate yield (11% starting from menadione) [148]. Additionally, chemical approaches have been used to synthetize novel vitamin K2 analogues, reported to have higher bioactivities when compared with their natural counterparts [136,149,150,151]. These new compounds, with vitamin K activity but different pharmacological properties compared with the natural homologues, might reveal novel and interesting biological activities for commercialization as active diet supplements.

Compared to chemical synthesis, biosynthetic production methods for natural vitamin K2 using bacterial fermentation have been the most studied and reported production systems for this vitamer [152,153,154]. This is mainly due to the advantage of selective production of the all-trans isomer by microorganisms, and the easy manipulation and culture conditions optimization of many bacterial strains. Over recent years, research on vitamin K2 biotechnological production has moved from the identification of bacterial types producing K2, to screening of K2 high-producing bacterial strains, often combined with genetic mutations and resistant mutants leading to improved K2 yields. More recently, bioengineered K2 metabolic pathways using high-producing bacterial strains and improved culture conditions have been described. 

Biotechnological strategies using either liquid and solid state fermentation processes (LSF and SSF), and modifications in culture conditions such as media composition and carbon source, temperature, shaking speed, and time in culture, have been developed for vitamin K2 production in several bacterial types such as *Flavobacterium* sp., Lactic acid bacteria, *Bacillus subtilis*, *Bacillus subtilis natto, Bacillus amyloliquefaciens,* and *Bacillus licheniformis* (reviewed in [155]). In general, the highest levels of vitamin K2 are produced by *Bacillus* species. Since *Bacillus subtilis* has been granted the status of GRAS [156], and several methods to improve vitamin K2 bacterial productivity have resulted in high yield, these are positioned among the most important industrial vitamin K producers for its use as diet supplements providing human health benefits. Particularly, *Bacillus subtilis natto* has been shown to produce a range of vitamin K2 homologues (MK-4, MK-5, MK-6, MK-7, and MK-8) with the major component being MK7 and accounting for more than 90% of total vitamin K2 production [157]. Depending on the strategy employed, *Bacillus subtilis natto* has been reported to produce MK-7 with yields of 3.6 mg/L in a mutant strain resistant to 1-hydroxy-2-naphthoic acid (HNA) [158], 32.2 mg/L in an isolated strain from the traditional Japanese food natto fermented for 72 h [159], 35.0 mg/L in a menadione-resistant mutant strain isolated from natto cultivated for four days [160], and 1719 μg/100 g natto [161] in a multiple resistant mutant strain. More recently, approaches of metabolic engineering to enhance MK-7 production in *Bacillus subtilis* have been reported [162]. This strategy is based on the overexpression of different combinations of rate-limiting enzymes involved in MK-7 biosynthetic pathways. Using these approaches in the *B. subtilis* 168 strain, yields of 50 mg/L [163] and 69.5 mg/L [164] of MK-7 were reported. This represents a considerable improvement on vitamin K2 production, and further optimization may open the perspective for new and affordable manufacturing processes allowing a considerable reduction of costs that will benefit the final consumer. 

### 5.3. Aquatic Organisms as Sources of Vitamin K with a Potential Biotechnological Application

In recent years, an increasing interest is given to aquatic organisms, as a source of useful and sustainable bioproducts meeting the increasing market and consumer demands for nutritional supplements, with benefits in human health promotion and disease prevention [165]. Freshwater and marine organisms, especially macroalgae, microalgae, and several species of cyanobacteria, have been widely recognised as a valuable source of diet supplements and functional ingredients with great health benefits [166,167]. In this area, a considerable amount of research efforts has been focused on improving the biomass supply and bioactive extraction by developing safe, sustainable, and environmentally-friendly processes [168].

The ability to synthesise vitamin K has been already described for several marine organisms such as macroalgae, microalgae, and cyanobacteria. In particular, the synthesis of vitamin K1 is reported in different species of macroalgae and microalgae such as *Porphyra* sp. (Rhodophyta), *Sargassum muticum*, *Sargassum fusiforme*, *Undaria pinnatifida*, *Nannochloropsis oculata* (Ochrophyta), *Tetraselmis suecica*, *Dunaliella salina*, *Desmodesmus asymmetricus*, *Chlorella vulgaris*, *Chlamydomonas reinhardtii* (Chlorophyta), *Isochrysis galbana*, *Pavlova lutheri* (Haptophyta), and *Skeletonema costatum* (Bacillariophyta) [121,122,169,170]. Additionally, several species of cyanobacteria are described to be able to biosynthesise and produce vitamin K1 such as *Anabaena cylindrica*, *Anabena variabilis*, *Spirulina* sp., and *Nostoc muscorum, Synechocytis* sp. PCC 6803 [124,171]. Biosynthesis of phylloquinone has been mainly associated with oxygenic photosynthetic organisms such as plants, algae, and cyanobacteria. MKs are described to be synthetized by a limited number of obligate and facultative anaerobic bacteria. Nevertheless, several species of cyanobacteria and microalgae, such as the cyanobacteria *Gloeobacter violaceus* [172] and *Synechococcus* sp. PCC 7002 [173], the diatom *Chaetoceros gracilis* [174], and the red algae *Cyanidium caldarium* [175] have been shown to synthetize MK-4. Moreover, evidence from the literature describes several aquatic species with different content in vitamin K1 [169]. This suggests that algae should be an attractive and potential source of biomass for biosynthesis of vitamin K production with potential for biotechnological applications [146]. In fact, vitamin K1 has been found in variable amounts in several species of macroalgae, microalgae, and cyanobacteria, as summarized in Table 2. In a recent study analysing seven different microalgae species, the cyanobacteria *Anabaena cylindrica* was identified as the richest source of the active E-isomer of vitamin K1 (200 μg/g dry weight) [170] (Table 2). This concentration was around six times higher when compared with parsley (*Petroselinum crispum*) [170], which is a known rich dietary source of K1, and higher than any other previously reported phylloquinone dietary source [115,175,176,177]. Additionally, the method proposed in this study uses low temperatures, low pressures, and sustainable feedstocks, precluding great prospects for biotechnological and industrial application. Furthermore, the cyanobacteria *Spirulina* sp. was found to have a content of 0.255 μg/g dry weight [170], and, in the marine green microalgae *Tetraselmis suecica,* the concentration of vitamin K1 on a dry weight basis was 28 µg/g [169].

Within macroalgae, two species of edible seaweeds native to Japan, *Sargassum muticum* and *Undaria pinnatifida* are considered highly invasive species with negative economic and ecological impacts for the region [178,179]. Both macroalgae species were tested as biomass for the extraction and quantification of vitamin K1 and shown to have different contents with 12.9 µg/g dry mass in *Undaria pinnatifida* [121] as well as a remarkably high content in *Sargassum muticum* (750 µg/g dry matter) [169]. The exploitation of this biomass as a vitamin K1 source should encourage its harvesting and control, bringing a potential economic and environmental interest. 

Macroalgae species have been described as a potential alternative for the biosynthetic production of vitamin K1 due to its higher vitamin K1 content when compared to terrestrial plants. On the other hand, microalgae and cyanobacteria represent an interesting alternative for the biotechnological industrial production of vitamin K1 due to their easy cell manipulation when compared with plant cell cultures. Another advantage of using microalgae for industrial production of products intended for human consumption is the fact that many species are considered as GRAS.

## 6. Vitamin K Formulations and Impact on Absorption and Bioavailability 

Recently, a few studies have addressed the production of novel and more stable vitamin K formulations to improve vitamin K absorption and bioavailability. This is of crucial importance for patients with cholestasis due to extremely low level of bile salts in the intestine. Konakion^®^ (phytomenadione) mixed micelles (MM) is a formulation composed of phytomenadione in clear bile acid/lecithin MM solution, for oral or parenteral administration, used for the prophylaxis and treatment of VKDB [180]. Nevertheless, some reports describe that these formulations do not increase vitamin K bioavailability because they are unstable and tend to aggregate in gastric pH conditions [181]. Strategies to increase its stability, absorption, and bioavailability include mixed formulations with poly (ethylene glycol) [182] and saponins [183], which are preferred components due to its natural plant origin (such as quillaja bark and soybean) and consist of a hydrophobic polycyclic aglycone tail, attached to one or more saccharide moieties. Saponin-containing Konakion^®^ MM were shown to be a promising oral formulation for vitamin K due to its increased stability at low pH, cytocompatibility, and cell uptake capacity [183].

Intramuscular administration of vitamin K1, although effective, has raised concerns related with the administration, such as injection pain, skin bruising, and toxic ingredients, which compromise therapy compliance [183,184]. Recently, an innovative drug delivery mechanism was developed as an alternative to the hypodermic conventional needles drug administration, consisting of drug-loaded microneedles (MNs). MNs matrix or baseplate are impregnated with the required molecule/drug. After dermic application, the interstitial fluid is able to dissolve the MNs and allow the content release, which represents a micro-dimensional and less invasive method. Several substances were already successfully delivered using this system by enabling the transdermal delivery of drugs that can be absorbed directly into the systemic circulation [185,186,187,188]. A recent study was developed to investigate the production of microneedles for the delivery of vitamin K [189]. The in vitro results showed that vitamin K in a microneedle array was successfully delivered in neonatal porcine skin over 24 h. This methodology, even though it still needs to be clinically validated, anticipates great potential for improvement of patient compliance in vitamin K prophylaxis in developed countries and might contribute to reduce VKDB cases in undeveloped countries [189].

## 7. Conclusions

Overall, the concept of multifunctional vitamins associated with vitamin K has been growing in recent decades with evidence showing its involvement in a wide range of biological functions with a pivotal role in several highly prevalent low-grade inflammatory diseases. Several age-related diseases such as skeletal and CVD, Alzheimer’s disease, and dementia are becoming a major social and economic burden in our aging society. Compelling clinical evidence combined with a strong scientific biological rational clearly support a beneficial health effect of vitamin K and has led to an increased procurement of vitamin K as a health promoting supplement. Interestingly, some scientific evidence from in vitro and in vivo models, as well as from clinical studies, suggests a synergistic effect of vitamin K combined with vitamin D, with beneficial effects of joint supplementation at optimal concentrations of both vitamins, particularly for bone health [190,191]. However, although the benefits of vitamin D in bone health are well established, high levels of vitamin D might promote hypercalcemia and soft tissue calcification with consequent detrimental effects on the cardiovascular system [192]. While additional studies are required to establish the optimal concentration of a combined supplementation with vitamins K and D, high levels of K1, MK4, or MK7 have no documented toxicity or adverse health effect. No hypercoagulable state was observed in individuals consuming doses above the recommended daily allowance of 75 micrograms vitamin K (Commission Directive 2008/100/EC) [193]. Additional specific cases of extremely high levels of vitamin K intake have also been reported without adverse effects [194,195]. However, the current and increasing knowledge on the different types of vitamin K vitamers and their specific biological activity imply a clearer differentiation between the potential health effect and target specificity for each vitamer. It is well accepted that both K1 and K2 can play an important role in the pathogenesis and progression of many diseases. Nevertheless, the K2 vitamer (MK-7) has been shown to have advantages given its superior bioavailability and higher half-life in circulation when compared with other K vitamers. In addition, the vast majority of available clinical studies are still related to the effects of vitamin K1 in health, while K2 has been shown to have a prevalent function in extra-hepatic tissues with a protective role in the vascular system reducing the risk of CVD, mitigating cognitive diseases, and suppressing inflammation. Although both vitamin K1 and K2 are commercially available, optimized production methods and more efficient formulations for each vitamer are needed to meet the increasing customer requirements at affordable prices. Additionally, marine diet supplements and functional products are already well represented in the global market and the exploitation of new aquatic-derived sources for vitamin K should represent a benefit for human health with a potential economic and environmental interest.

## Figures and Tables

**Table 1:** Vitamin K-dependent proteins.

	Designation	Function
**Hepatic**	Factor II (Prothrombin)	Pro-coagulant [7]
	Factor VII	Pro-coagulant [7]
	Factor IX	Pro-coagulant [7]
	Factor X (Stuart Factor)	Pro-coagulant [7]
	Protein C	Anti-coagulant, anti-inflammatory, anti-apoptotic [7,8,9,10]
	Protein S	Co-factor for activated protein C, anti-coagulant, bone turnover, anti-inflammatory [7,8,11,12]
	Protein Z	Regulation of coagulation, anti-thrombotic [13,14]
**Extra Hepatic**	OC	Negative regulator of bone formation, regulator of mineral maturation rate, mechanical stabilizer of bone matrix, regulator of glucose metabolism [15,16]
	MGP	Inhibitor of soft tissue calcification, modulator of angiogenesis and tumorigenesis [17,18,19]
	Gas6	Signal transduction, regulator of proliferation, migration, differentiation, adhesion, and apoptosis, anti-inflammatory, platelet activation, thrombus stabilization [20,21,22]
	GRP	Inhibitor of soft tissue calcification, inhibitor of mineral crystal maturation and growth in blood, anti-inflammatory [23,24,25,26]
	Periostin (isoforms 1–4)	Regulator of cell-matrix interactions, adhesion, proliferation, and differentiation processes, tissue remodelling and wound repair, angiogenesis [27,28,29]
	PRGP1/PRGP2	Signal transduction [30,31]
	TGM3/TGM4	Signal transduction [32]
**Endoplasmic Reticulum/Golgi Apparatus**	GGCX	γ-carboxylation of VKDPs [33]

VKDPs, Vitamin K-dependent Proteins. OC, Osteocalcin. MGP, Matrix Gla-Protein. Gas6, growth arrest-specific protein-6. GRP, Gla-Rich Protein. PRGP, proline-rich Gla protein. TGM, transmembrane Gla protein. GGCX, γ-glutamyl carboxylase.

**Table 2:** Vitamin K1 content in algae.

	Phylum	Species Designation	Content (µg/g)
**Macroalgae**	Ochrophyta	*Undaria pinnatifida* *^1^	12.9 [121]
		*Sargassum fusiforme* *^2^	1.75 [121]
		*Sargassum muticum*	750 [169]
	Rhodophyta	*Porphyra* sp. *^3^	26 [122,169]
**Microalgae**	Bacillariophyta	*Skeletonema costatum*	5.5 [169]
	Chlorophyta	*Tetraselmis suecica*	28 [169]
		*Dunaliella salina*	0.1 [170]
		*Desmodesmus asymmetricus*	0.46 [170]
		*Chlorella vulgaris*	0.73 [170]
	Cyanobacteria	*Anabaena cylindrica*	200.25 [170]
		*Spirulina* sp.	12.70 [170]
	Haptophyta	*Isochrysis galbana*	8 [169]
		*Pavlova lutheri*	6.5 [169]
	Ochrophyta	*Nannochloropsis oculata*	0.17 [171]

*^1^ Also known as Wakame. *^2^ Also known as Hijiki. *^3^ Also known as Nori.

## Abbreviations

CHD	Coronary heart disease
CKD	Chronic kidney disease
CRP	C-reactive protein
CVD	Cardiovascular disease
DOACs	Direct oral anticoagulants
GGCX	γ-glutamyl carboxylase
Gas6	Growth arrest-specific protein 6
Gla	γ-carboxyglutamic acid
Glu	Glutamic acid
GRAS	Generally recognized as safe
GRP	Gla-rich protein
HD	Haemodialysis
IKB	IkappaB kinase
INR	International normalized ratio
KH2	Vitamin K hydroquinone
KO	Vitamin K 2,3-epoxide
LDL	Low-density lipoproteins
12-LOX	12-lipoxygenase
LPS	Liposaccharide
LSF	Liquid-state fermentation process
MGP	Matrix-Gla protein
MKs	Menaquinones
MM	Mixed micelles
NF-κB	Nuclear factor kappa B
OA	Osteoarthritis
OC	Osteocalcin
PRGP1	Proline-rich Gla proteins
RA	Rheumatoid arthritis
ROS	Reactive oxygen species
SSF	Solid-state fermentation process
TLR	Triacylglycerol-rich lipoproteins
TMG	Transmembrane Gla proteins
VC	Vascular calcification
VKAs	Vitamin K antagonists
VKDB	Vitamin K deficiency bleeding
VKDPs	Vitamin K-dependent proteins
VKORC1	Vitamin K epoxide reductase complex subunit 1
VKORC1L1	Vitamin K epoxide reductase complex subunit 1-like 1
WHO	World Health Organization
